# CRISPR-Cas9 Mediated Mutation in *OsPUB43* Improves Grain Length and Weight in Rice by Promoting Cell Proliferation in Spikelet Hull

**DOI:** 10.3390/ijms23042347

**Published:** 2022-02-21

**Authors:** Qi Wu, Yingfan Liu, Junli Huang

**Affiliations:** Key Laboratory of Biorheological Science and Technology, Ministry of Education, Bioengineering College, Chongqing University, Chongqing 400044, China; wuqilv181114@yeah.net (Q.W.); yingfanliu@yeah.net (Y.L.)

**Keywords:** rice, grain size, OsPUB43, BR, OsMADS6, OsMADS34, cell proliferation

## Abstract

Grain weight, a crucial trait that determines the grain yield in rice, is influenced by grain size. Although a series of regulators that control grain size have been identified in rice, the mechanisms underlying grain development are not yet well understood. In this study, we identified OsPUB43, a U-box E3 ubiquitin ligase, as an important negative regulator determining the gain size and grain weight in rice. Phenotypes of large grain are observed in *ospub43* mutants, whereas overexpression of *OsPUB43* results in short grains. Scanning electron microscopy analysis reveals that *OsPUB43* modulates the grain size mainly by inhibiting cell proliferation in the spikelet hull. The OsPUB43 protein is localized in the cytoplasm and nucleus. The *ospub43* mutants display high sensitivity to exogenous BR, while *OsPUB43*-OE lines are hyposensitive to BR. Furthermore, the transient transcriptional activity assay shows that OsBZR1 can activate the expression of *OsPUB43*. Collectively, our results indicate that OsPUB43 negatively controls the gain size by modulating the expression of BR-responsive genes as well as MADS-box genes that are required for lemma/palea specification, suggesting that OsPUB43 has a potential valuable application in the enlargement of grain size in rice.

## 1. Introduction

Rice (Oryza *sativa* L.), as one of the major yield crops in the world, provides food for more than half of the world’s population [1,2,3,4]. How to sustainably increase rice yields is a future research direction to prevent global food shortages [5,6,7,8]. The yield in rice is determined by three important agronomic traits: the grain weight, grain number per panicle, and panicle number per plant [5,9,10], and grain weight is determined by multiple factors, such as grain length, grain width, grain thickness, and grain filling rate [10,11]. Studies in Arabidopsis and rice indicated that seed size is modulated by multiple signaling pathways and regulators, including the HAIKU (IKU) pathway, the ubiquitin–proteasome pathway (UPP), phytohormones signaling, mitogen-activated protein kinase (MAPK) signaling, G-protein signaling (RGS), and numerous transcriptional regulatory factors [11,12,13,14,15]. Despite recent advances, the exact mechanisms underlying seed size still remain to be elucidated.

The ubiquitin-proteasome system demonstrated an important yet multifaceted role in many cellular processes in eukaryotes, such as responses to various abiotic and biotic stresses, and hormone signaling and response [16,17,18,19,20]. Protein ubiquitination is mediated by three types of enzymes: ubiquitin-activating enzyme (E1), ubiquitin-conjugating enzyme (E2), and ubiquitin ligase (E3) [21,22,23]. Based on their subunit composition and mechanisms of action, E3 ubiquitin ligases have be classified into four groups: HECT, RING, U-box, and cullin RING ligases (CRLs), with the CRLs further divided into four subtypes [24]. The U-box E3 ubiquitin ligases that come from a modified RING finger motif, were discovered first in yeasts and ubiquitously present across eukaryotic organisms. As for plant U-box (PUB) ligases, Arabidopsis thaliana contains 64 U-box E3 ubiquitin ligases [25,26], while the monocotyledonous model crop rice has 77 PUB proteins [23]. 

Recent studies indicated that the ubiquitin-proteasome pathway is a key manner for seed size control [12,13,14,15]. The ubiquitin receptor DA1 acts maternally to control seed size by restricting the integument cell proliferation in Arabidopsis [27,28,29]. Two E3 ubiquitin ligases, DA2 and EOD1/BB, both of which physically interact with DA1, act synergistically to restrict organ and seed size [27,28,29]. Furthermore, the ubiquitin-specific protease UBP15, one of substrates of DA1, is a positive regulator of cell proliferation and promotes ovule integument growth, thus controlling seed size in Arabidopsis [30]. Numerous studies demonstrated that maternal plant can influence seed size by affecting the integument cell proliferation [13,29,30,31]. Similar to the restraint of the integuments in the dicot plant Arabidopsis, the spikelet hull limits the seed development space and restricts grain size in rice [13]. Various ubiquitin-related regulatory factors that affect the growth of spikelet hull were identified in rice [12,13,15,32]. For instance, the GW2-WG1-OsbZIP47 signaling cascade regulates grain width and weight by modulating the cell proliferation in the spikelet hull in rice [33]. WG1 is ubiquitinated by the E3 ubiquitin ligase GW2 and degraded via the ubiquitin pathway, and therefore releases the OsbZIP47 transcription factor and activates the expression of downstream development-related genes [33,34,35]. In another report, the ubiquitin receptor HDR3 forms a complex with GW6a, and the transgenic rice plants overexpressing *HDR3* and *GW6a* have a larger grain than the wild type, indicating that both of them play a positive role in the spikelet hull cell proliferation [36,37]. OsUBP15 (Ubiquitin-Specific Protease 15) acts as an active regulator of grain width and size, and the dominant gain-of-function rice mutant *lg1-D* of OsUBP15 shows the enhanced cell proliferation in the spikelet hull [38]. Although the ubiquitin-proteasome pathway plays critical roles in modulating grain size, their precise functions remain largely unknown in monocotyledonous plants.

Plant hormone brassinosteroids (BRs) have essential functions in regulating grain size in rice, and generally positively regulate the grain size [39,40]. The BR-insensitive mutant *osbak1* shows small grain, and OsBAK1 regulates the development of spikelet hull by affecting cell proliferation in rice [41]. Consistently, the BR biosynthesis deficient mutant *d11* shows the reduced grain size [42]. Moreover, the *cpb1* mutant, a *d11* allelic mutant, has reduced grain size and erect-leaf phenotype [43]. Supportively, overexpression of *D11* in the *cpb1* mutant, which is an allelic mutant of *D11*, not only recovers normal panicle architecture and plant height, but also increases the leaf angle and grain size. The mutant *d61-7* that harbors a mutation in BR receptor gene *OsBRI1* exhibits a reduction of grain size [44]. DLT acts as a positive regulator of BR signaling, and GSK2 negatively regulates BR response [45]. *DLT* overexpression lines show increased grain length, while *GSK2* overexpression lines have short grain [46]. 

To date, extensive studies have shown that the ubiquitin-proteasome pathway and BR signaling pathway work together to influence the grain size [47,48]. The rice E3 ubiquitin ligase TUD1 is involved in the growth regulation via BR pathway, and the length of grain hull is severely limited in the *tud1* mutants [47]. The *elf1* mutant was a visibly shorter and smaller grain than the wild type [48], which is similar to BR-deficient mutant *brd2* (*BR-deficient dwarf2*) and BR receptor mutant *d61* (brassinosteroid insensitive1, *osbriI*), indicating that the E3 ubiquitin ligase ELF1 plays a critical role in BR signaling in rice [39,49,50,51].

The classical ABCDE model that determines the identity of floral organs generated from studies of dicots can also be applied to explain the floral development of grasses such as rice [52]. In Arabidopsis, the MADS-box genes of class E are called SEPALLATA (SEP), and the homologues genes which are required for the development of spikelet and flower in rice are *OsMADS1*, *OsMADS5*, *OsMADS6*, *OsMADS7*, *OsMADS8,* and *OsMADS34* [53,54]. Notably, *OsMADS1*, *OsMADS6*, and *OsMADS34* play a crucial regulatory role in the development of lemma/palea [53,54], which was verified by the genetic evidence. The loss of *OsMADS1* results in larger and flatter cells than that in the wild type, but overexpression of *OsMADS1* causes increased cell number in lemma [55]. Rice *afg1*, an allele mutant of *OsMADS6*, has a shorter grain and lower grain quality than the wild type [56]. Grains on secondary branch of *osmads34-t* is short, compared with wild type [57]. The *OsMADS1^lgy3^*, which is a natural variant of *OsMADS1* due to alternatively splicing, is overexpressed in Nipponbare background and transgenic rice produces a longer grain than Nipponbare [58].

In this study, we generated *ospub43* mutants by CRISPR-Cas9 system, and the mutants produced large and heavy grains. On the contrary, overexpression of *OsPUB43* caused short and light grains. Meanwhile, the expression levels of BR-responsive genes, such as *DLT* and *OsBZR1*, were lower in *OsPUB43*-OE lines than that in the wild type, suggesting that OsPUB43 might regulate grain development by influencing BR response in rice. In addition, OsPUB43 could modulate spikelet hull development by influencing the expression of multiple MADS-box genes and cell cycle genes. Thus, our findings defined OsPUB43 as a critical factor that determined grain size and weight, suggesting its potential value in increasing rice yield.

## 2. Results

### 2.1. OsPUB43 Functions as a Negative Regulator of Grain Size and Weight

To reveal the regulatory function of U-box E3 ubiquitin ligase in the seed development in rice, we analyzed the spatio-temporal expression profile for a subset of U-box E3 ubiquitin ligase genes by the PlaNet and GENEVESTIGATOR tools [59,60], found that *OsPUB43* (LOC_Os02g34410) was preferentially expressed in the inflorescence or panicle (Figure S1). The analysis from PlaNet showed abundant expression of *OsPUB43* in inflorescence during the rice development, especially in P1–P5 inflorescence developmental stages (Figure S1), suggesting its essential roles in the spikelet hull development. In support of our hypothesis, the development of the rudimentary and empty glumes of spikelet hull took place during the early inflorescence growth stage [61,62]. The result from GENEVESTIGATOR also indicated that *OsPUB43* was highly expressed in spikelet cell and pollen (Figure S2). Together, these analyses suggested the important functions of OsPUB43 in the development of spikelet hull. 

**Figure 1:**
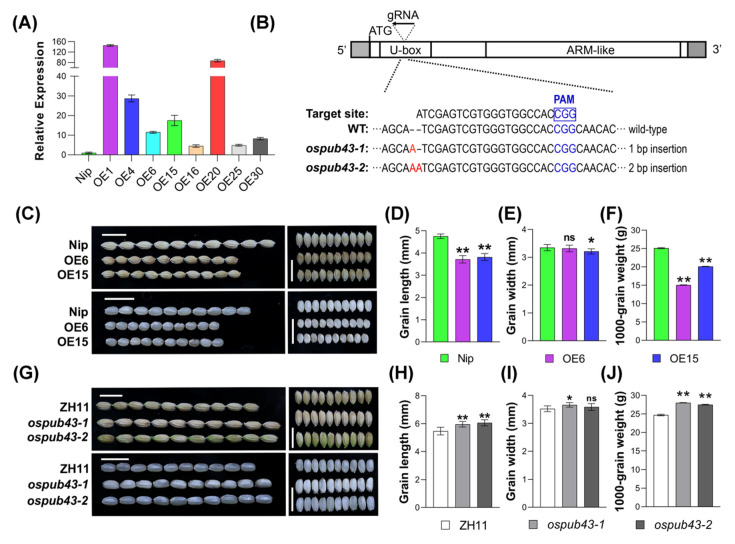
OsPUB43 is a negative modulator of grain length and weight in rice. (**A**) Relative expression levels of *OsPUB43* in the leaf of eight independent overexpression lines. *Actin1* was used as the endogenous control. Relative expression values represent the means ± SE from three biological replicates (*n* = 3). (**B**) Schematic diagram of intron-free *OsPUB43* gene structure and single-guide RNA (sgRNA)-targeted sites. UTRs and exons are indicated by gray boxes and white boxes, respectively. These insertions introduced premature termination codon, which produced a 149 or 198 amino acid truncation. (**C**,**G**) Mature grains of wild type (Nip, ZH11), *OsPUB43*-OE lines and *ospub43* mutants. Scale bars= 1 cm. (**D**–**F**, **H**–**J**) Comparisons of grain length, grain width, and 1000-grain weight of wild type, *OsPUB43*-OE lines and *ospub43* mutants. Student’s *t*-test is used to generate the *p*-values in (**D**–**F**, **H**–**J**). Bars with different letters are significantly different at ** p < 0.01 or * p < 0.05. ns, not significant.

We obtained multiple independent transgenic rice lines overexpressing OsPUB43 (Figure 1A). To further evaluate the function of OsPUB43, we then also produced the *ospub43* mutants (*ospub43-1* and *ospub43-2*) by CRISPR/Cas9 system (Figure 1B and Figure S3). Mutant *ospub43-1* has a one-nucleotide insertion, and *ospub43-2* has a two-nucleotide insertion in the position of 142 bp after ATG, respectively, which lead to frameshift mutations and premature stop codons, producing truncated proteins (Figure 1B and Figure S3). The observation of grain size showed that the grains of multiple *OsPUB43* overexpression (*OsPUB43*-OE) lines were shorter and narrower than that of wild type (Nip) (Figure 1C–E; Figure S4A,B). Statistical analysis indicated that the grain length of *OsPUB43*-OE lines was reduced by 21.8% and 19.7%, whereas the grain width of most transgenic lines was reduced significantly (Figure 1D,E; Figure S4B,C). We also examined the influence of OsPUB43 in grain weight, an important agronomy trait. As shown in Figure 1F, overexpression of *OsPUB43* markedly reduced the 1000-grain weight in two *OsPUB43*-OE lines, with 40.2% and 20.0% reduction, in comparison with the non-transgenic grains. We then investigated the grain size and weight in two independent knockout mutants (*ospub43-1* and *ospub43-2*). In contrast to *OsPUB43* overexpression lines, knockout of *OsPUB43* resulted in increased grain length, and the grains of mutants were 9.0% and 10.9% longer than non-transgenic seeds (ZH11) (Figure 1G,H). There was no significant difference in grain width between *ospub43* mutants and non-transgenic seeds (Figure 1I). In support of the increased grain length, the grain weight was also significantly enhanced, with 10.9% and 7.0% elevation compared to non-transgenic seeds (Figure 1J). These results provided evidence that OsPUB43 served as a negative regulator in grain development and thus reduced weight regulation in rice.

### 2.2. OsPUB43 Restricts Grain Development by Inhibiting Cell Proliferation in Spikelet Hull

**Figure 2:**
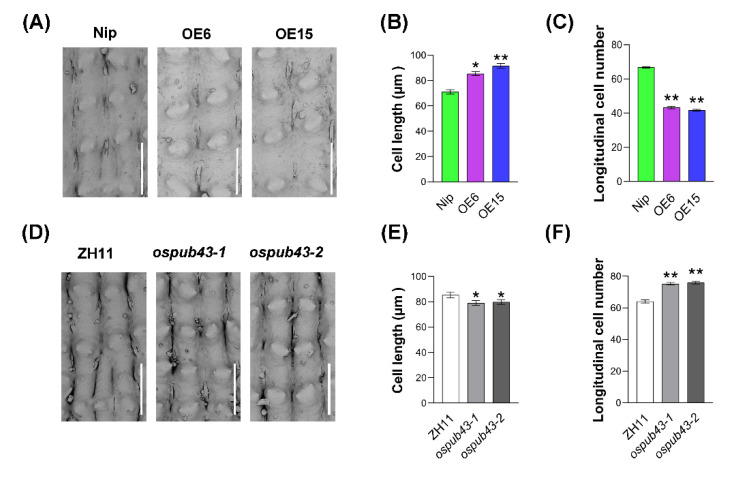
OsPUB43 controls grain size through its negative role in the regulation of cell number in spikelet hulls. (**A**,**D**) Scanning electron microscopic analysis of the outer surfaces in the spikelet hulls of wild type (Nip, ZH11), *OsPUB43*-OE lines and *ospub43* mutants. Scale bars = 100 μm. (**B**,**E**) Outer epidermal cell length in the spikelet hulls along the grain length orientation in wild type, *OsPUB43*-OE lines and *ospub43* mutants. (**C**,**F**) Outer epidermal cell number in the spikelet hulls in wild type, OsPUB43-OE lines and *ospub43* mutants. Student’s *t*-test is used to generate the *p*-values in (**B**,**C**,**E**,**F**). Bars with different letters are significantly different at ** p < 0.01 or * p < 0.05.

Seed size is dictated by the signal integration of maternal and zygotic tissues, and the spikelet hull limited the grain development in rice [13]. To investigate OsPUB43 inhibition on the grain development whether by limiting the cell proliferation or cell expansion in spikelet hull, we investigated the cell size as well as number in the outer lemma of spikelet hulls by scanning electron microscopy. Unexpectedly, compared to Nip, the cell length in *OsPUB43*-OE spikelet hulls in the grain length direction was significantly increased, while the cell number in the grain length direction was greatly reduced (Figure 2A–C). These results demonstrated that OsPUB43 restricted grain development by inhibiting cell proliferation in spikelet hull, thus resulting in reduced cell number in the grain length direction. We further investigated the cell length and cell number of spikelet hulls in the *ospub43* mutants. Supportively, the cell number of spikelet hulls in *ospub43* mutants was significantly increased compared with that in the ZH11, although the cell expansion was slightly inhibited (Figure 2D–F), suggesting that the enlarged spikelet hulls of *ospub43* mutants were caused by the increased cell number, not the cell length. These results demonstrated that OsPUB43 restricted grain development by affecting cell proliferation and thus influencing the cell number in the spikelet hull.

### 2.3. Subcellular Localization and Expression of OsPUB43

**Figure 3:**
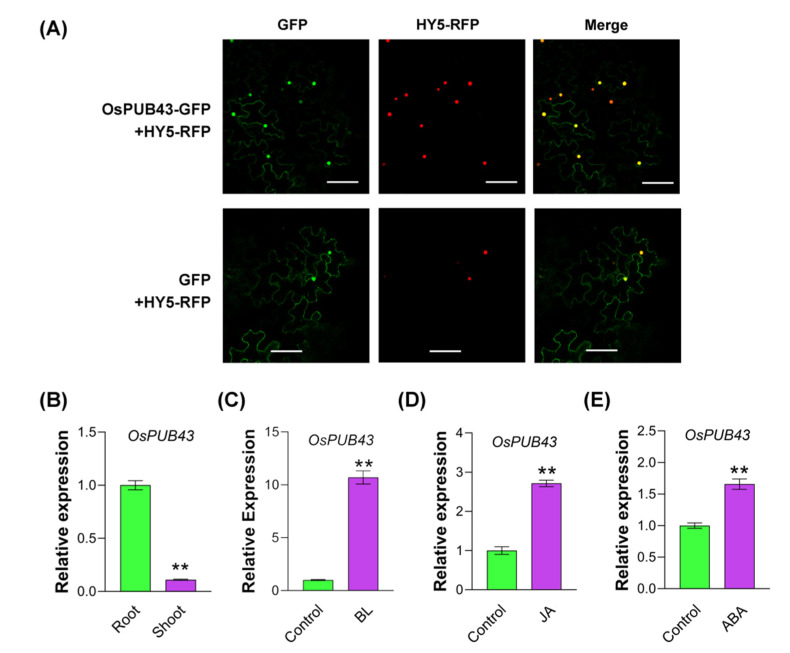
Subcellular localization and expression of OsPUB43. (**A**) Subcellular localization of OsPUB43. The fusion construct (*35S:OsPUB43-eGFP*) was transformed into tobacco (*Nicotiana benthamiana*) epidermal cells respectively, via Agrobacterium tumefaciens-mediated transformation. The empty vector (*35S: eGFP*) was used as a control. GFP images were taken. The HY5-RFP was used as a nuclear marker. The merged images are shown on the right. Scale bars = 100 μm. (**B**) Expression levels of endogenous *OsPUB43* in the shoot and root. Total RNAs were extracted from the shoot and root of two-week-old rice seedlings. (**C**–**E**) Expression levels of endogenous *OsPUB43* after BL, JA, and ABA treatment. Total RNAs were extracted from the root of 2-week-old rice seedlings after treatment. In (**B–E**), values are means ± SE. Data were analyzed using Student’s *t*-test. Bars with different letters are significantly different at ** p < 0.01.

To investigate the subcellular localization of OsPUB43, OsPUB43 was fused with eGFP, and driven by the CaMV *35S* promoter, and GFP fluorescence signals were detected by laser scanning confocal microscopy in the leaf epidermal cells of *Nicotiana benthamiana*. Consistent with the GFP fluorescence signals, the OsPUB43-eGFP was also localized in the nucleus and cytoplasm (Figure 3A), indicating that the OsPUB43 was a nuclear and cytoplasmic localized protein. 

*OsPUB43* was predominantly expressed in the root at seedling stage (Figure 3B). Subsequently, we detected whether *OsPUB43* expression was induced by phytohormones that were involved in regulating grain development. In rice, BRs positively regulate grain size, and BR-insensitive or BR-deficient mutants produce small seeds [41,63,64]. Hence, we detected that the transcript levels of *OsPUB43* were significantly up-regulated under the treatment with BL (Brassinolide) (Figure 3C). JA (Jasmonate) signaling could regulate flower organ development by influencing the expression of MADS-box genes [65,66]. We then examined the response of *OsPUB43* to exogenous MeJA (Methyl Jasmonate), and the result showed that MeJA could strongly induce the expression of *OsPUB43* (Figure 3D). In addition, ABA (Abscisic Acid) plays a negative role in grain size through reducing the endosperm cell numbers [12,67]. Our result showed the significantly up-regulated expression of *OsPUB43* in response to ABA (Figure 3E). These results indicated that the *OsPUB43* transcription was regulated by multi-hormone signaling pathway.

### 2.4. OsPUB43 Regulates the Rice Growth via the BR Signaling 

**Figure 4:**
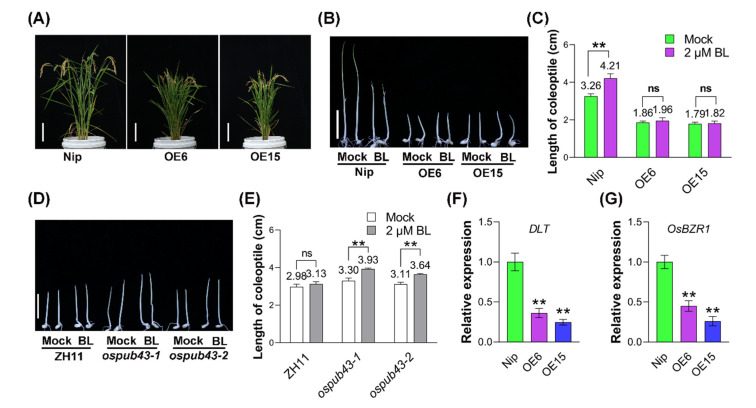
OsPUB43 negatively regulates the rice growth via the BR signaling. (**A**) Morphological phenotypes of 4-month-old overexpressing rice lines *OsPUB43*-OE-6 and -15, and the corresponding control (Nipponbare, Nip). Bars = 15 cm. (**B**,**D**) Coleoptile elongation response to 2 μM BL. (**C**,**E**) Statistical data for coleoptile length described in (**B**,**D**). (**F**,**G**) Expression levels of BR-responsive genes (DLT and OsBZR1) in panicles of the overexpressing rice lines *OsPUB43*-OE-6 and -15, and the corresponding control (Nipponbare, Nip). In (**C**,**E**–**G**), values are means ± SE. Data are analyzed using Student’s *t*-test. Bars with different letters are significantly different at ** p < 0.01.

The *OsPUB43*-OE lines exhibited a classic BR-deficient phenotype with a semi-dwarf plant and erect leaves, compared with Nip (Figure 4A), and also showed a typical dn-type pattern of internode elongation [68] (Figure S5), which was similar to that of the BR biosynthesis defective mutant *lhdd10* [69]. However, *ospub43* mutants showed the increased lamina joint bending angle (Figure S6), which is similar to the enhanced BR signaling phenotype of *Osbzr1-D* [70]. Thus, we asked whether OsPUB43 was involved in BR responses or BR biosynthesis. Given that *OsPUB43* was highly expressed in coleoptile (Figure S2), we examined the sensitivity of *OsPUB43*-OE lines, *ospub43* mutants and their corresponding wild type to exogenous BR by the coleoptile elongation assay. Uniformly germinated seeds were grown on 1/2MS medium supplemented with or without 2 μM BL in dark for 7 days. Comparison of coleoptile length showed that the growth of the coleoptile was promoted by exogenous BL more greatly in wild type plants than that in *OsPUB43*-OE transgenic lines (OE6 and OE15) (Figure 4B,C), suggesting that *OsPUB43*-OE lines were less sensitive than wild type. Notably, the hyposensitivity of *OsPUB43*-OE to BR was similar to the BR-insensitive *d61-2* mutants [71]. 

Furthermore, the BR sensitivity of *ospub43* mutants and ZH11 was tested. As expected, our results showed that the relative elongation of coleoptiles under BL treatment was greater in *ospub43* mutants than that in ZH11 (Figure 4D,E), indicating that loss of *OsPUB43* promoted plant sensitivity to BR. Together, these data demonstrated that BR response was significantly inhibited in *OsPUB43*-OE lines, while enhanced in *ospub43* mutants, hinting that OsPUB43 might restrict grain development by inhibiting BR signaling in rice.

To further investigate whether OsPUB43 regulated grain development by repressing BR signaling, we examined the mRNA levels of BR-responsive genes in the panicles of Nip and *OsPUB43*-OE lines by qRT-PCR analysis. *DLT* and *OsBZR1* have been reported as critical genes in BR signaling pathway in rice, and the *DLT*-OE or *OsBZR1*-OE plants show increased grain size while *OsBZR1*-RNAi lines act oppositely, exhibiting reduced grain length [40,46]. As we expected, the expression of *DLT* as well as *OsBZR1* was down-regulated in the *OsPUB43*-OE lines relative to Nip (Figure 4F,G), indicating a reduced BR response during the panicle development in the *OsPUB43*-OE lines. These observations suggested a close positive correlation between the BR response and grain size, which was consistent with previous reports [41,44,46,72].

### 2.5. OsPUB43 Modulates Spikelet Hull Development by Influencing the Gene Expression of Multiple MADS-Box Genes and Cell Cycle Genes

**Figure 5:**
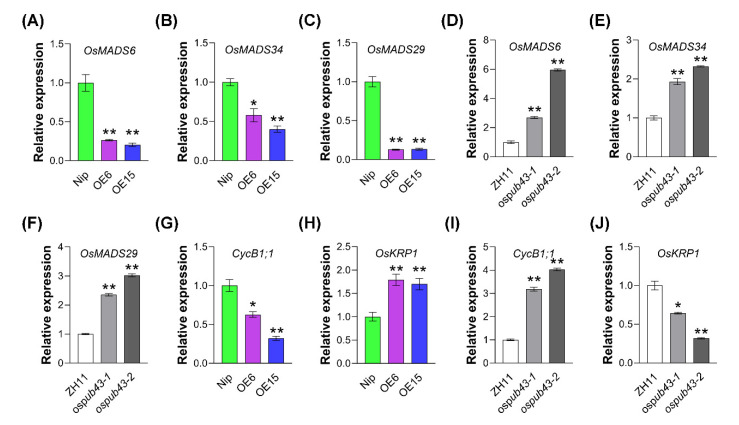
The expression of SEPALLATA (SEP) subfamily MADS-box genes and cell cycle genes are influenced by OsPUB43. (**A**–**F**) Expression levels of *OsMADS6*, *OsMADS29* and *OsMADS34* in panicles of the overexpressing rice lines *OsPUB43*-OE-6 and -15, *ospub43* mutants and the corresponding control (Nip, ZH11). (**G**–**J**) Expression levels of cell cycle regulate gene (*CycB1;1* and *OsKRP1*) in panicles of the overexpression rice lines *OsPUB43*-OE-6 and -15, *ospub43* mutants and the corresponding control. In (**A**–**J**), values are means ± SE. Data are analyzed using Student’s *t*-test. Bars with different letters are significantly different at ** p < 0.01, * p < 0.05.

MADS-box proteins have been well recognized to play essential roles in plant spikelet hull development [52,53,54,73]. To reveal the possible mechanisms underlying OsPUB43 modulating spikelet hull development, we detected the expression of *OsMADS6* and *OsMADS34* in the panicles of *OsPUB43*-OE lines, and the results showed that the mRNA levels of the two MADS-box genes were significantly lower in the panicles of *OsPUB43*-OE lines than that in Nip (Figure 5A,B). *OsMADS29* was indicated to regulate the seed development, and *osmads29* mutants produced shorter grain than wild type [74]. Consistently, the *OsMADS29* transcript level was significantly reduced in the panicles of *OsPUB43*-OE lines (Figure 5C). In contrast to *OsPUB43* overexpression lines, we further found the transcription levels of *OsMADS6*, *OsMADS29* and *OsMADS34* were significantly up-regulated in the *ospub43* mutants (Figure 5D–F). These results indicated that OsPUB43 might influence spikelet hull development by inhibiting the expression of *OsMADS6*, *OsMADS29* and *OsMADS34* in the panicles.

The BR signaling pathway is involved in the regulating cell division and cell expansion [75,76]. Accordingly, the reduced BR response in *OsPUB43*-OE lines urged us to hypothesize that the alteration of cell proliferation in spikelet hull of *OsPUB43*-OE lines and *ospub43* mutants might be caused by the changed expression of cell cycle genes. We thus detected the expression levels of cell cycle relative genes in the *OsPUB43*-OE lines and its wild-type counterpart. The results illustrated that expression of *CycB1;1*, a positive regulator of the cell cycle, was reduced in *OsPUB43*-OE lines (Figure 5G). Conversely, the mRNA abundance of *OsKRP1,* a gene for cyclin-dependent kinase inhibitor, increased in *OsPUB43*-OE lines (Figure 5H). On the contrary, the expression of *CycB1;1* was up-regulated and OsKRP1 was down-regulated in *ospub43* mutants (Figure 5I,J). These results confirmed that OsPUB43 could restrict cell division by regulating a cluster of cell cycle genes.

### 2.6. OsBZR1 Binds to OsPUB43 Promoter and Promotes Its Expression

**Figure 6:**
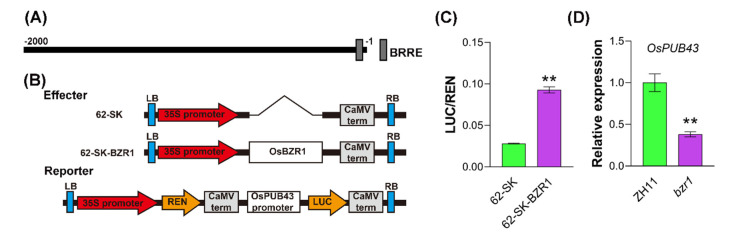
OsBZR1 binds to *OsPUB43* promoter and promotes its expression. (**A**) Schematic representation of the *OsPUB43* promoter structure. BRRE, BR response element. (**B**) Diagrams of the reporter and effector constructs. (**C**) OsBZR1 trans-activated *OsPUB43* by binding its promoter region. The ratio of LUC/REN of the empty vector (62-SK) was considered to be a control. The activation is indicated by the ratio of LUC to REN. (**D**) Expression levels of *OsPUB43* in seedling root of *osbzr1* mutant and the corresponding control (ZH11). In (**C**, **D**), values are means ± SE of three independent repeats. Data are analyzed using Student’s *t*-test. Bars with different letters are significantly different at ** p < 0.01.

OsBZR1 has been well recognized as a key transcription factor controller in BR signaling in rice [70,77,78,79]. To explore whether the *OsPUB43* was a target gene of OsBZR1, we analyzed the promoter sequence of *OsPUB43* and found a putative BRRE motif (Figure 6A). To further determine the DNA binding ability of OsBZR1 and its influence on the transcription of *OsPUB43* in vivo, the dual-luciferase reporter system was used. The luciferase gene in the pGreenII 0800-LUC vector was under control of the *OsPUB43* promoter fragment, and the fusion vector was used as a reporter construct (Figure 5B). The CDS of *OsBZR1* driven by *35S* promoter in pGreenII 62-SK vector was used as the effector construct and the empty vector served as control (Figure 6B). The reporter was co-transformed with the effector or the empty vector into four-week-old *Nicotiana benthamiana* leaves with an efficient agroinfiltration expression system, then the LUC activity was obtained based on LUC/REN ratio. As shown in Figure 6C, the LUC/REN ratio was drastically increased in *N. benthamiana* leaves containing OsBZR1 together with *OsPUB43_Pro_:LUC*, indicating that OsBZR1 can activate LUC expression driven by *OsPUB43* promoter in vivo. Consistently, the expression of *OsPUB43* was greatly reduced in *osbzr1* mutant, compared to that in the wild type (Figure 6D). These results demonstrated that *OsPUB43* acted as a target of transcriptional activator OsBZR1.

## 3. Discussion

Regulation of seed size is a fundamental question in developmental biology. Although multiple important modulators and various signaling pathways have been proposed [12,41,56,80], our understanding of how plants determine their seed size is still limited. Here, we identified OsPUB43 as a negative regulator of grain size and weight. Overexpression of *OsPUB43* resulted in the reduction of grain size and weight; on the contrary, knockout of *OsPUB43* increased the grain size and weight (Figure 1). In rice, the spikelet hull is a maternal limitation of the grain development, and its size is determined by cell proliferation and cell expansion in maternal tissues [12,35,81]. In our study, we also provided convincing evidence that OsPUB43 restricted grain length and weight by inhibiting cell proliferation in spikelet hulls (Figure 2). Consistent with its suppressive function in cell proliferation, *OsPUB43* was predominantly expressed in the spikelet during the development of inflorescence (Figures S1 and S2). Thus, our findings identified *OsPUB43* as an important factor that modulated grain size in rice, offering a strategy for enlarging grain size and increasing yield.

It has been reported that BRs promote cell expansion and cell proliferation, and positively affect grain size, resulting in enhancement of grain size [5,78,82]. Mutation of *D2*, *D11*, *D61* and *OsBAK1* leads to the phenotype of small grain [41,42,44,50,72], while overexpression of *DLT* in rice results in large grains [46]. Overexpression of *OsPUB43* suppressed the BR response (Figure 4B,C); however, loss of *OsPUB43* enhanced the BR signaling (Figure 4D,E). These changes of BR response in *OsPUB43*-OE lines prompted us to examine the BR related gene expression in the transgenic rice panicles. Here, we found that *OsPUB43* overexpression resulted in significant decrease of the *DLT* and *OsBZR1* expression in the panicles (Figure 4F,G). These results clearly demonstrated that OsPUB43 played a negative role in BR responses and OsPUB43 might restrict the grain development of *OsPUB43*-OE lines through inhibition of the BR signaling.

The large grain phenotype of *ospub43* mutants resulted from a large number of cells might be due to an increased cell division rate (Figure 2 and Figure 5B), which was closely associated with cell cycle regulation. BR response deficiency in the *OsPUB43*-OE lines was likely to be directly responsible for the reduced cell division, which was similar to the function of rice *qGL3* in suppressing BR signaling and cell division in spikelet hulls [78,82]. Consistent with this notion, the expression of the cell cycle positive regulator *CycB1;1* was down-regulated and the cell cycle negative regulator *OsKRP1* was up-regulated in the *OsPUB43*-OE lines (Figure 5G,H). The results demonstrated that OsPUB43 can influence cell cycle genes expression to regulate the cell proliferation in spikelet hull, at least partially through inhibiting BR signaling.

Recent studies implicated that the spikelet development is regulated by a list of OsMADS-box proteins, such as OsMADS1, OsMADS6, OsMADS15, OsMADS17 and OsMADS34 [52,53,56,57,83]. Previous studies demonstrated that OsMYC2, a key transcriptional activator in JA signaling, regulates the expression of *OsMADS1*, *OsMADS7,* and *OsMADS14*, suggesting the JA signaling pathway is closely associated with the floral meristem identity specification [84,85,86,87]. Moreover, OsMYC2 also can bind the promoter of *OsMADS4*, *OsMADS5*, *OsMADS8* and *OsMADS34* in Y1H assay [86]. *OsMADS34*, which determines the lemma/palea identity together with *OsMADS1* [88], plays an essential role in determination of grain size in rice, and *osmads34-t* mutants exhibit small grains on its secondary branches of panicles [57]. *OsMADS6* plays an important role in determining palea identity and affecting grain yield and quality in rice by controlling the cell proliferation [56]. The expression levels of *OsMADS6*, *OsMADS29* and *OsMADS34* were reduced in *OsPUB43*-OE lines and increased in *ospub43* mutants (Figure 5A–F). Our study suggested that OsPUB43 could inhibit a subset of OsMADS-box genes to restrict the development of lemma/palea.

**Figure 7:**
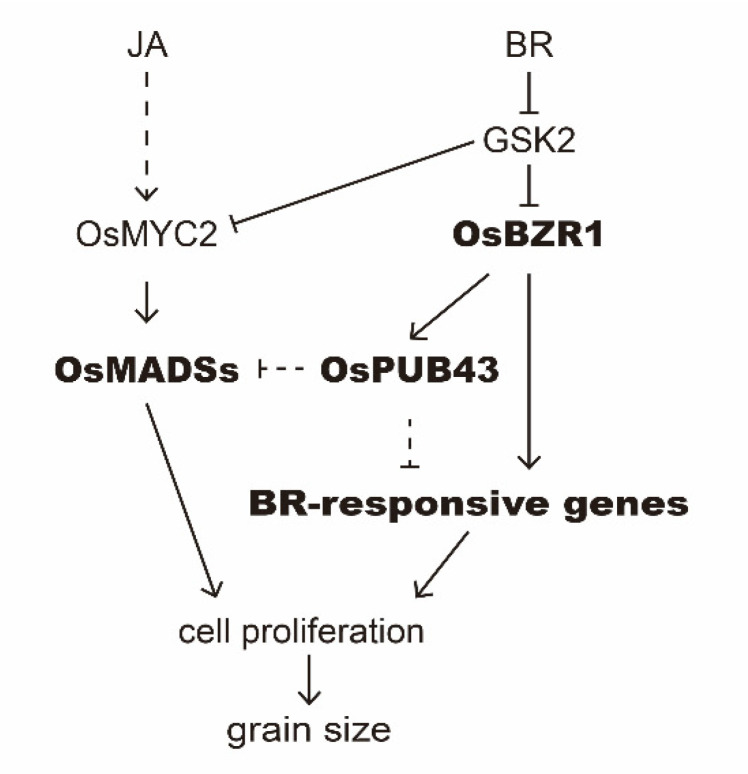
A proposed working model for OsPUB43-mediated signaling pathways in rice. OsPUB43 acts as a repressor of the OsMADS-dependent signaling pathway and the BR signaling pathway, contributing to limitation to cell proliferation. In *OsPUB43* overexpression lines, the BR-responsive genes and OsMADS-box genes are reduced, and cell proliferation is inhibited, resulting in short grain. In *ospub43* mutants, the expression of OsMADS-box genes is increased, and cell proliferation is promoted, resulting in large grain.

In addition, as a key negative regulator of BR signaling, OsGSK2, which participated in the grain development [46,89,90,91], physically interacts with OsMYC2, resulting in the degradation of OsMYC2 by phosphorylation [92]. Based on these results, we propose a working model to elucidate how OsPUB43 functions as a negative regulator in grain development (Figure 7). In wild type, BRs inhibit the activity of GSK2, which releases the OsBZR1 to active the transcription of BR response gene [45,46,90,93]. On the other hand, OsBZR1 also increase the expression level of *OsPUB43*, which in turn represses the BR response in rice. In *ospub43* mutants, knockout of *OsPUB43* alleviated the inhibition of OsPUB43 on BR response, thus resulting in the promoted BR signaling output and increased grain size. BR homeostasis is important for the growth of plant, and the negative regulator OsPUB43 may modulate dynamics of BR response in rice. In addition to BR response, the expression of *OsMADS6* and *OsMADS34* is very important for the development of rice lemma and palea, and they are activated by MYC2 but inhibited by OsPUB43. Collectively, our results indicate that OsPUB43 function as a repressor of BR signaling in cell proliferation and works as an important negative regulator to maintain homeostasis of cell proliferation. The approach may have potential in rice molecular breeding for high yield.

## 4. Materials and Methods

### 4.1. Plant Material and Growth Conditions

Rice (*Oryza sativa* ssp. japonica) cv Nipponbare (Nip) and cv Zhonghua 11 (ZH11) were used in this study. For constructing *OsPUB43*-OE transgenic plants, the coding sequence (CDS) of *OsPUB43* was cloned into pCAMBIA1301-eGFP, driven by *35S* promoter. Mutants of *OsPUB43* were generated by CRISPR/Cas9 system as described previously [94]. These constructs were introduced into Nip and ZH11 by *Agrobacterium tumefaciens*-mediated transformation, respectively. Loss-of-function mutant *osbzr1* (RMD_04Z11PM21) was identified from RMD mutant database of ZH11 [95]. The plants were grown in the field from April to October under natural conditions in Chongqing, China, or in the greenhouse under a 14 h: 10 h, light: dark photoperiod with 60% humidity in winter. The primers were listed in Table S1.

### 4.2. Subcellular Localization of OsPUB43

The full-length coding sequence of OsPUB43 from Nipponbare, lacking the stop codon, was amplified and cloned upstream of the eGFP coding region in the pCAMBIA-eGFP vector under the control of CaMV *35S* promoter to generate the plasmid *OsPUB43*-eGFP. To study the subcellular localization of OsPUB43, *OsPUB43*-eGFP plasmid and control eGFP vector were introduced into 4-week-old *Nicotiana benthamiana* leaves by *Agrobacterium tumefaciens*-mediated transformation, respectively. After incubation for 48 h, GFP fluorescence was observed by Leica SP8 confocal microscope. HY5-RFP was used as a nucleal location maker. The primers are listed in Table S1.

### 4.3. Plant Hormone Treatment

The BR sensitivity test method was performed as described previously [63,71]. The seeds were sterilized by 5% sodium hypochlorite and grown in the 1/2 Murashige and Skoog (MS) medium containing 0 or 2μM BL, then the coleoptile lengths were measured after 1 week growth in darkness. The length of coleoptiles was measured by analyzing digital images using ImageJ software (1.47v, NIH, Bethesda, MD, USA).

For the phytohormone treatments, seeds were germinated in distilled water for 4 days, then placed in Yoshida solution with for another 10 days. They were then treated with MeJA (100 μM), BL (50 μM), or abscisic acid (100 μM) for 1 h.

### 4.4. RNA Isolation, Reverse Transcription, and Quantitative Real-Time PCR Assays

RNA isolation was performed using a TRIzol-based method for Plant RNA extraction [96,97], and reverse transcription was performed using an PrimeScript™ RT reagent Kit (Takara, Japan) with gDNA Eraser. 2× TSINGKE Master qPCR Mix (SYBR Green I with UDG) (TsingKe Biotech Co., TSE203, Beijing, China) was used for quantitative real-time PCR with the Bio-Rad CFX 96 (Bio-Rad, Hercules, CA, USA). Gene expression level was normalized using rice *Actin1* (LOC_Os03g50885) as a reference gene. The primers are listed in Table S1.

### 4.5. Morphological and Cellular Observation

For cell size measurements, mature grains were scanned using the Hitachi SU3500 (Hitachi, Tokyo, Japan) scanning electron microscope after gold spraying treatment, and original images of the 50× field and 300× field were obtained. The outer epidermal cells in the central part of the lemmas (300× field) were measured using ImageJ (1.47v, NIH, Bethesda, MD, USA), and the cell number was counted in the grain length direction (50× field).

### 4.6. Accession Numbers

Sequence data from this article can be found in the rice genome annotation project databases under the following accession numbers: *OsPUB43* (LOC_Os02g34410); *DLT* (LOC_Os06g03710); *OsBZR1* (LOC_Os07g39220); *OsMADS6* (LOC_Os02g45770); *OsMADS29* (LOC_Os02g07430); *OsMADS34* (LOC_Os03g54170); *CycB1;1* *(LOC_Os01g59120)*; *OsKRP1 (LOC_Os02g52480)*; *Actin1 (LOC_Os03g50885)*. 

## Data Availability

The data will be available from the corresponding author upon requeset.

## Supplementary Materials

The following supporting information can be downloaded at: https://www.mdpi.com/article/10.3390/ijms23042347/s1.
