# Supplementary material for: CRISPR-Cas9 Mediated Mutation in OsPUB43 Improves Grain Length and Weight in Rice by Promoting Cell Proliferation in Spikelet Hull
Source: Int J Mol Sci. 2022 Feb 21;23(4):2347. doi: 10.3390/ijms23042347 (PMC8877319; doi:10.3390/ijms23042347)
Supplement: Supplementary file 1 [file ijms-23-02347-s001.zip › ijms-1591373-Supplementary Figures.pdf]

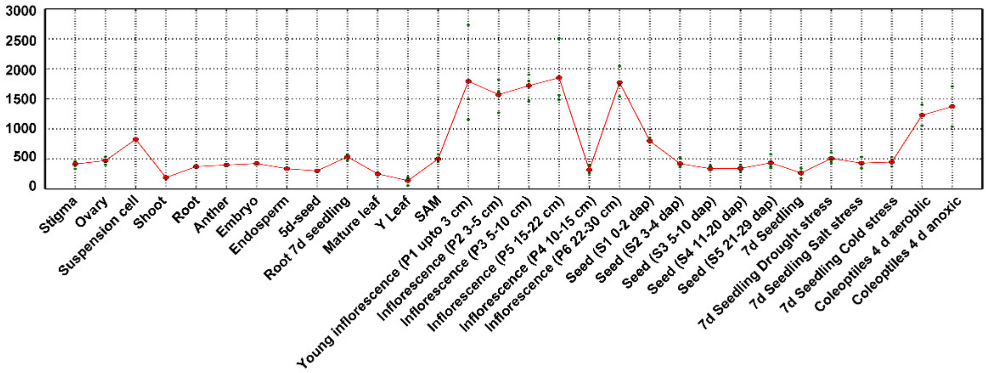

Figure S1 Spatio-temporal expression profile of *OsPUB43*. The expression profile of *OsPUB43* is analyzed by using the PlaNet Browser.

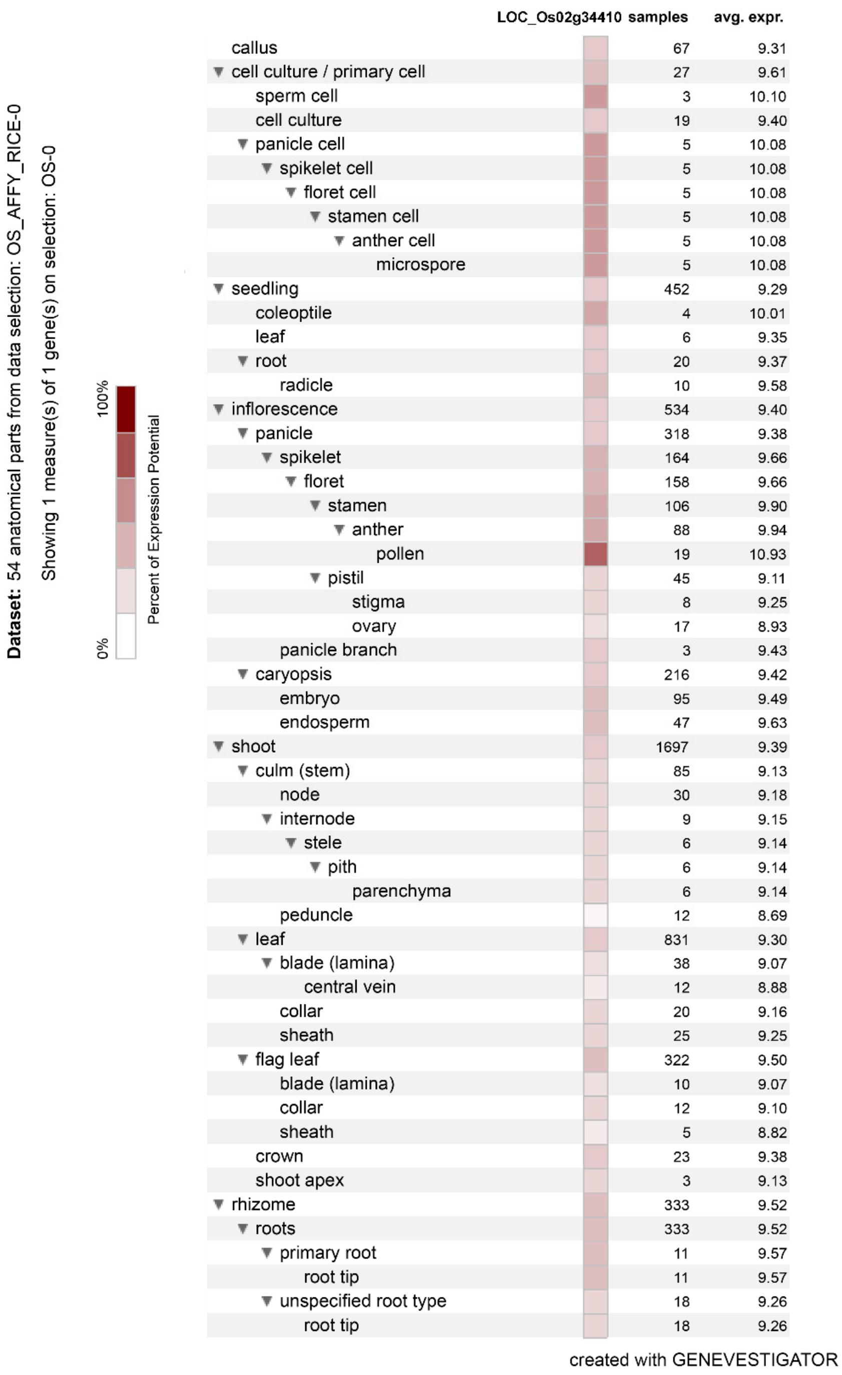

Figure S2 Spatio expression profile of *OsPUB43*. The expression profile of *OsPUB43* is analyzed by using the Genevestigator.

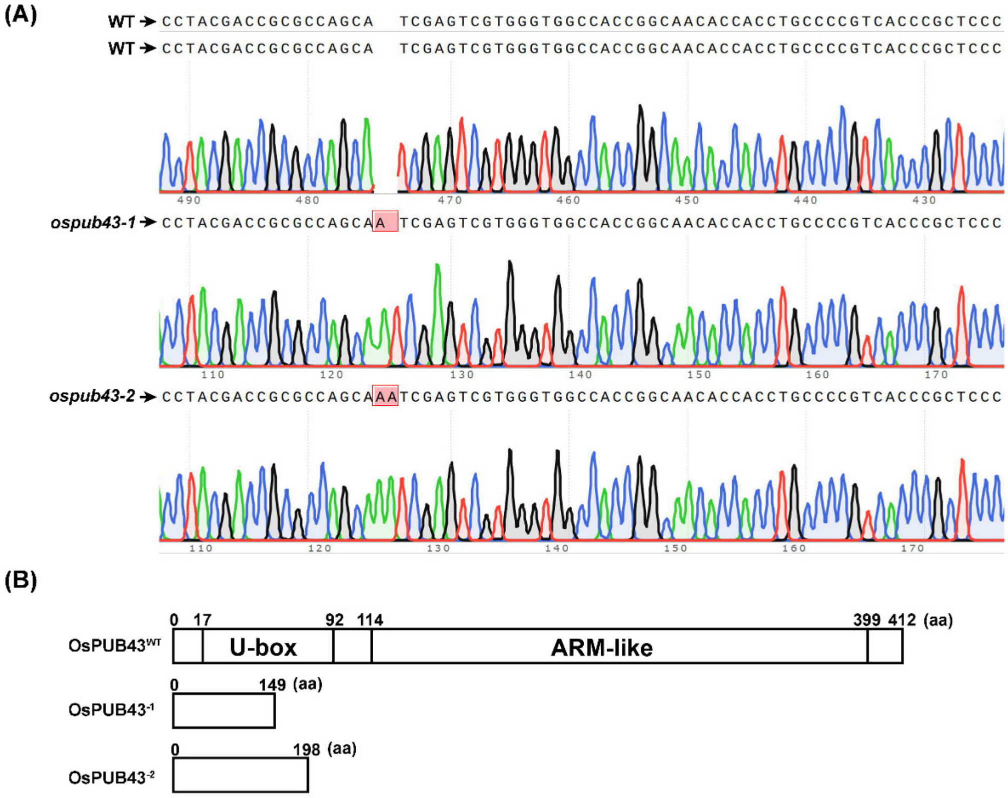

Figure S3 Generation of *ospub43* mutant alleles by CRISPR/Cas9 system. (A) DNA sequence chromatograms obtained from CRISPR/Cas9-derived T1 progeny, together with a wild-type control. (B) Predicted protein structures of *ospub43* alleles. OsPUB43^{-1} and OsPUB43^{-2} are composed of 149 and 198 amino acids, respectively

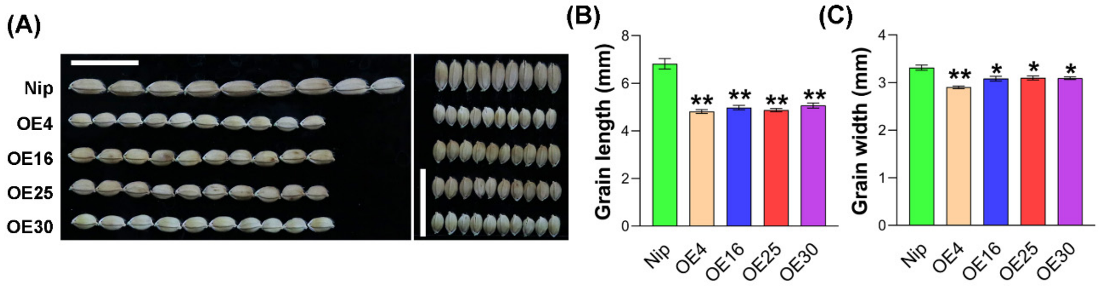

Figure S4 OsPUB43 is a negative modulator of grain length and width in rice.

(A) Mature grains of wild type (Nip) and *OsPUB43*-OE lines.

(B, C) Comparisons of grain length and width of wild type with that of *OsPUB43*-OE lines.

Student's *t*-test is used to generate the *P*-values in (B, C). Bars with different letters are significantly different at $^{**}P < 0.01$ or $^{*}P < 0.05$.

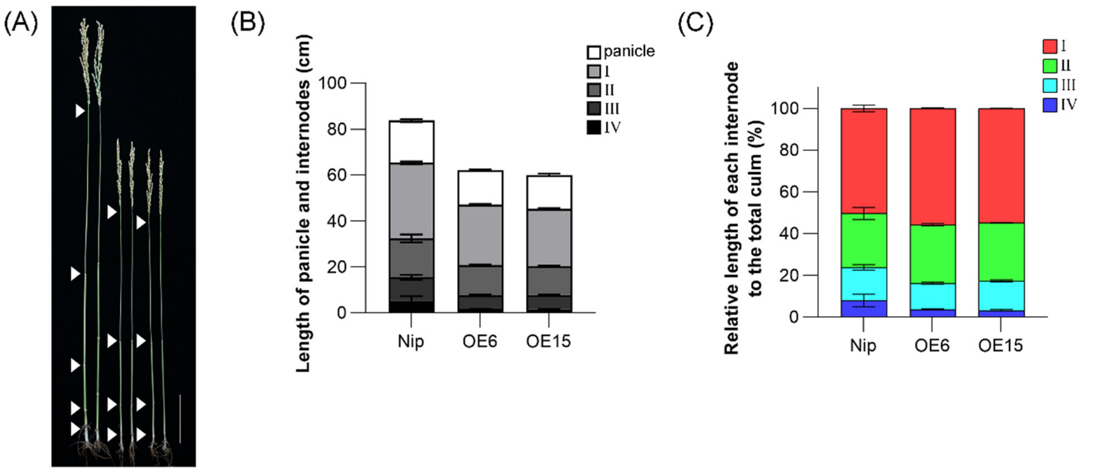

Figure S5 Rice *OsPUB43*-OE lines display shorter basal internodes than wild type (Nip).
(A) Nip and *OsPUB43*-OE lines phenotypes of culm (4 months old). Scale bars, 10 cm.
(B) Internode length of Nip and *OsPUB43*-OE lines culm. Values are means ±SE (n= 10 plants).
(C) Length of the internode as a percentage of the total internode length. Numbers indicate internodes from the uppermost to the fourth.

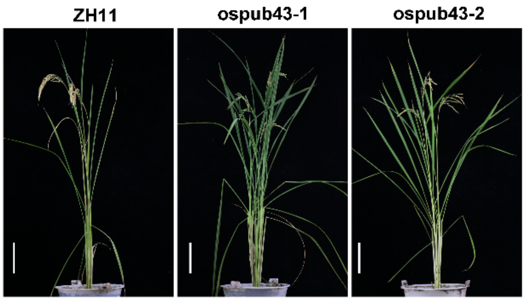

Figure S6 Morphological phenotypes of 4-month-old mutant lines *ospub43-1* and *ospub43-2*, and the corresponding wild type (Zhonghua11, ZH11). Bars = 15 cm.
